# Identification of diagnostic biomarkers and immune cell profiles associated with COPD integrated bioinformatics and machine learning

**DOI:** 10.1111/jcmm.70107

**Published:** 2024-09-30

**Authors:** Zirui Zhu, Zhuo Zeng, Baichen Song, Huishan Chen, Huiqing Zeng

**Affiliations:** ^1^ Department of Pulmonary and Critical Care Medicine Zhongshan Hospital of Xiamen University, School of Medicine, Xiamen University Xiamen China; ^2^ National Institute for Data Science in Health and Medicine Xiamen University Xiamen China; ^3^ Xiamen University Tan Kah Kee College Zhangzhou China

**Keywords:** bioinformatics, COPD, gene biomarkers, immune infiltration, machine learning

## Abstract

This retrospective transcriptomic study leveraged bioinformatics and machine learning algorithms to identify novel gene biomarkers and explore immune cell infiltration profiles associated with chronic obstructive pulmonary disease (COPD). Utilizing an integrated analysis of metadata encompassing six gene expression omnibus (GEO) microarray datasets, 987 differentially expressed genes were identified. Further gene ontology and pathway enrichment analyses revealed the enrichment of these genes across various biological processes and pathways. Moreover, a systematic integration of two machine learning algorithms along with pathway‐gene correlations identified six candidate biomarkers, which were validated in a separate cohort comprising six additional microarray datasets, ultimately identifying ADD3 and GNAS as diagnostic biomarkers for COPD. Subsequently, the diagnostic efficacy of ADD3 and GNAS was assessed, and the impact of their expression levels on overall survival was further evaluated and quantified in the validation cohort. Examination of immune cell subtype infiltration found increased proportions of cytotoxic CD8^+^ T cells, resting and activated NK cells, along with decreased M0 and M2 macrophages, in COPD versus control samples. Correlation analyses also uncovered significant associations between ADD3 and GNAS expression and infiltration of various immune cell types. In conclusion, this study elucidates crucial COPD diagnostic biomarkers and immune cell profiles which may illuminate the immunopathological drivers of COPD progression, representing personalized therapeutic targets warranting further investigation.

## 1 INTRODUCTION

Chronic obstructive pulmonary disease (COPD), characterized by irreversible airflow limitation, impacts both industrialized and developing regions as the third leading cause of death worldwide.^1^ Severe dyspnea and acute exacerbation are major causes of death in late‐stage COPD, thus early diagnosis of COPD is crucial, with numerous studies aiming to predict its severity to improve diagnostic and treatment strategies.^2^, ^3^, ^4^ Traditional spirometry, measuring the forced expiratory volume in 1 s and the forced vital capacity, remains the gold standard for diagnosing airflow limitation.^5^ While valuable, spirometry has limitations including variability, dependence on patient effort and inability to fully capture the complex nature of COPD. Computed tomography (CT) scans can supplement spirometry by revealing structural and functional lung changes, and identifying comorbidities or complications. They show potential for early COPD detection in individuals with respiratory symptoms but normal spirometry results.^6^ However, the lack of standardized criteria for interpreting CT scans and the risks of increased radiation exposure necessitate cautious use alongside other COPD diagnostic methods. Most importantly, developing new, efficient and non‐invasive techniques for early‐stage COPD diagnosis is urgently needed.

Recent advances in microarray technology and bioinformatics have enabled the identification of novel genes with significant diagnostic and prognostic potential. For instance, Yang et al. discovered that EXPH5 was specifically downregulated in late‐stage COPD using bioinformatics and machine learning, suggesting its potential as a novel biomarker.^7^ Esther et al. analysed sputum samples with a metabolomic mass spectrometric panel and found that elevated levels of sialic acid and hypoxanthine in COPD patients, correlating with disease severity and exacerbation risk.^8^ Zhong et al. discovered that the down‐expression of FHL1 might play a key role in cigarette smoke‐induced COPD using bioinformatics methods and peripheral blood mononuclear cells validations, proposing a regulatory mechanism involving the hsa‐miR‐664a‐3p and FHL1 axis.^9^ Overall, these genes are considered crucial potential biomarkers for COPD diagnosis.

In addition to gene changes, COPD causes varying degrees of immune dysfunction by affecting the structure and function of airways and alveoli.^10^, ^11^ Moreover, the condition of various immune cells changes as COPD progresses. For example, Jasper et al. demonstrated that neutrophils play a crucial role in COPD pathogenesis, driving pulmonary inflammation and representing potential therapeutic targets.^12^ Rao et al. summarized how Natural killer (NK) cell imbalances correlate strongly with COPD development, focusing on their biological characteristics.^13^ Benson et al. found that eosinophil distribution and activity within the lungs vary throughout the course of COPD and identified key clinical factors influencing this variation.^14^ In conclusion, immune cell function is pivotal in COPD and merits further investigation.

The recent integration of bioinformatics and machine learning offers a potent interdisciplinary approach for analysing large‐scale datasets, including microarray chips and transcriptome sequences. This method has significantly contributed to the development of early diagnosis for COPD.^3^, ^15^ In pursuing early diagnosis, many efforts have focused on developing innovative computer‐aided methods. For example, Yu et al. developed a reliable model for assessing preliminary COPD severity using a few lung sound channels with the Hilbert–Huang transform and ReliefF algorithm, demonstrating exceptional performance in classification.^16^ Shah‐Mohammadi et al. applied four machine learning models, combined with natural language processing of emergency room notes, to improve differential diagnosis in COPD patients.^17^ Despite these advancements, precise clinical diagnosis of COPD remains largely uncharted, underscoring the critical need for further research.

This study utilized a metadata cohort comprising six COPD microarray datasets from the gene expression omnibus (GEO) database to identify differentially expressed genes (DEGs) between COPD and controls using an innovative data inclusion method, followed by gene ontology (GO) enrichment, gene set enrichment analysis (GSEA), and the kyoto encyclopedia of genes and genomes (KEGG) pathway‐gene correlation analysis. Subsequently, we combined two machine learning algorithms with functional analysis to screen candidate biomarkers, which were validated in a separate cohort comprising six additional COPD microarray datasets. This process identified two final diagnostic biomarkers, ADD3 and GNAS. We then assessed their diagnostic efficacy in both the metadata and validation cohorts and quantified the impact of their expression levels on overall survival (OS) in the validation cohort.

Moreover, we employed the cell‐type identification by estimating relative subsets of RNA transcripts (CIBERSORT) algorithm to delineate immune cell differences between COPD and controls, and examined the correlation between two diagnostic biomarkers and immune cell infiltration. Collectively, our findings revealed two momentous genes that may hold promise for early diagnosis and management of COPD patients.

## 2 MATERIALS AND METHODS

### 2.1 Data sources

The gene expression data utilized in this study were sourced from the publicly accessible GEO databank (https://www.ncbi.nlm.nih.gov/geo/). We compiled a metadata cohort comprising six datasets, namely GSE8500, GSE38974, GSE63073, GSE71220, GSE76925 and GSE103174. Additionally, we used GSE8581, GSE20257, GSE37768, GSE47460, GSE106986 and GSE151052 as validation cohort. All datasets were selected based on their relevance to COPD and the quality of the data. The gene expression profiles across these series were normalized to standardized signal intensities. Normalization steps included background correction, log2 transformation and quantile normalization to ensure comparability across datasets.

### 2.2 Data pre‐processing and DEGs screening

We utilized the R language and corresponding annotation packages to pre‐process the microarray profile datasets, including platform records and series matrix files. The probe annotation files, containing probe names for each GSE series, were converted into standardized gene symbols. We addressed batch variability across platforms using the ‘combat’ function of the ‘SVA’ package. We initially identified DEGs using the Bioconductor ‘limma’ package with a consistent inclusion criterion of *p* < 0.05 across all datasets. For the metadata cohort, our goal was primarily to screen candidate biomarkers, and thus, based on the specific characteristics of each dataset, we applied a more lenient threshold for absolute log2 fold change (|log2FC|), set at ≥0.05 and ≥0.5. In contrast, for the validation cohort, our objective was to determine diagnostic biomarkers, so we adopted a more stringent |log2FC| threshold of ≥1 to ensure robust validation of our findings.

After data pre‐processing, DEGs were firstly visualized within the metadata cohort, since we included six GEO datasets, the traditional method of taking the intersection of all datasets might result in a large bias. Instead, we analysed DEGs in each dataset and implemented an innovative inclusion method, which has only been reported in a few studies,^18^, ^19^ that is, genes that appeared in the intersection of ≥3 datasets were considered as the inclusion objects. Lastly, we visualized the results based on our inclusion threshold using python.

### 2.3 Comprehensive function analysis

To further decipher the biological implications of the DEGs included in our study, we employed Metascape (http://metascape.org) database for GO enrichment analysis, considering GO terms with *p* < 0.05 as significantly enriched. We then utilized GSEA to identify enriched or depleted pathways among these DEGs, ranking all genes based on their differential expression between the two phenotypes using a *t*‐test. We computed an enrichment score for each gene set using the Hallmark Gene Sets from the molecular signatures database and the KEGG pathway database, reflecting the gene frequencies at the top or bottom of the ranked list. This analysis was conducted using GSEA software, visualizing the top three significant terms at *p* < 0.05.

Building on the GSEA results, we further refined our analysis by selecting genes with |log2FC| ≥0.05 or 0.5 from both COPD and control groups within the metadata cohort. These genes were then used for a detailed KEGG pathway‐gene correlation analysis. For this, we utilized the DAVID database (https://david.ncifcrf.gov/), Cytoscape software (version 3.10.2) as well as its associated plugins ClueGO^20^ and Cluepedia^21^ to conduct a comprehensive co‐relevant network of the KEGG terms together with pathways‐related genes.

### 2.4 Feature selection based on machine learning

To further conduct the feature selection among DEGs, we employed two machine learning algorithms: the least absolute shrinkage and selection operator (LASSO) and support vector machine‐recursive feature elimination (SVM‐RFE).

LASSO regression is a technique that facilitates both variable selection and regularization by reducing the coefficients of less crucial features to zero.^22^ In this study, we applied LASSO using the ‘glmnet’ package, employing a method of regression analysis that incorporated regularization for variable selection.

SVM‐RFE iteratively eliminates features by ranking them according to a SVM classifier. This widely used supervised machine learning technique for classification and regression identifies optimal variables by recursively removing those with the least impact on model performance, based on SVM‐generated eigenvectors.^23^ In this study, we implemented SVM‐RFE using the ‘e1071’ package.

Subsequently, we identified candidate biomarkers based upon a systematic integration of two distinct machine learning algorithms along with KEGG pathway‐gene correlation network for subsequent analysis. Following this, we utilized the validation cohort for further verification. Similar to our previously mentioned objects inclusion method, genes that appeared in the intersection of ≥3 datasets were considered as the final inclusion objects, thereby defining our diagnostic biomarkers.

### 2.5 Clinical utility of diagnostic biomarkers

Firstly, we standardized the data to ensure consistency and reliability due to the significant differences in gene expression across different datasets and utilized the nonparametric Kruskal–Wallis test to compare continuous variables between COPD and controls. To assess the diagnostic efficacy of the biomarkers within the metadata and validation cohorts, we plotted receiver operating characteristic (ROC) curves using the ‘pROC’ package. The Area Under the Curve (AUC) was calculated to quantify the diagnostic performance, with higher AUC values indicating better discriminatory ability between COPD and controls.

Subsequently, we further assessed the prognostic value of diagnostic biomarkers in the validation cohort using Kaplan–Meier survival estimates. These estimates, facilitated by the ‘survival’ and ‘survminer’ packages, allowed us to analyse and visualize the distribution of OS from the initiation of the study until death or last follow‐up. Survival curves were stratified based on the median expression levels of diagnostic biomarkers within the COPD cohort, defining high and low expression groups.

To quantify the impact of gene expression on survival while controlling for potential confounders, we employed the Cox proportional hazards model, implemented through the ‘survival’ package. This provided various indices and offered a deeper understanding of the roles of diagnostic biomarkers in COPD progression.

### 2.6 Evaluation of immune cell infiltration

To accurately evaluate immune scores, we employed the ‘immunedeconv’ package, and selected the CIBERSORT algorithm to analyse the cellular composition of complex tissues from gene expression profiles. Additionally, we used the ‘ggplot2’ package to visualize the differences between COPD and controls across 22 major immune cell types and to display the results of Pearson correlation analysis exploring the relationships between diagnostic biomarkers and infiltrating immune cells.

### 2.7 Statistical analysis

All statistical analyses were conducted using R software (version 4.2.3) and Python (version 3.8). Student's *t*‐test was employed for comparisons between two groups and one‐way ANOVA for multiple group comparisons. The non‐parametric Kruskal–Wallis test compared continuous variables between groups. The log‐rank test compared survival distributions between high and low expression groups. Associations between diagnostic biomarkers and immune cells were examined using Pearson correlation. Results with *p* < 0.05 were considered statistically significant.

## 3 RESULTS

**FIGURE 1:**
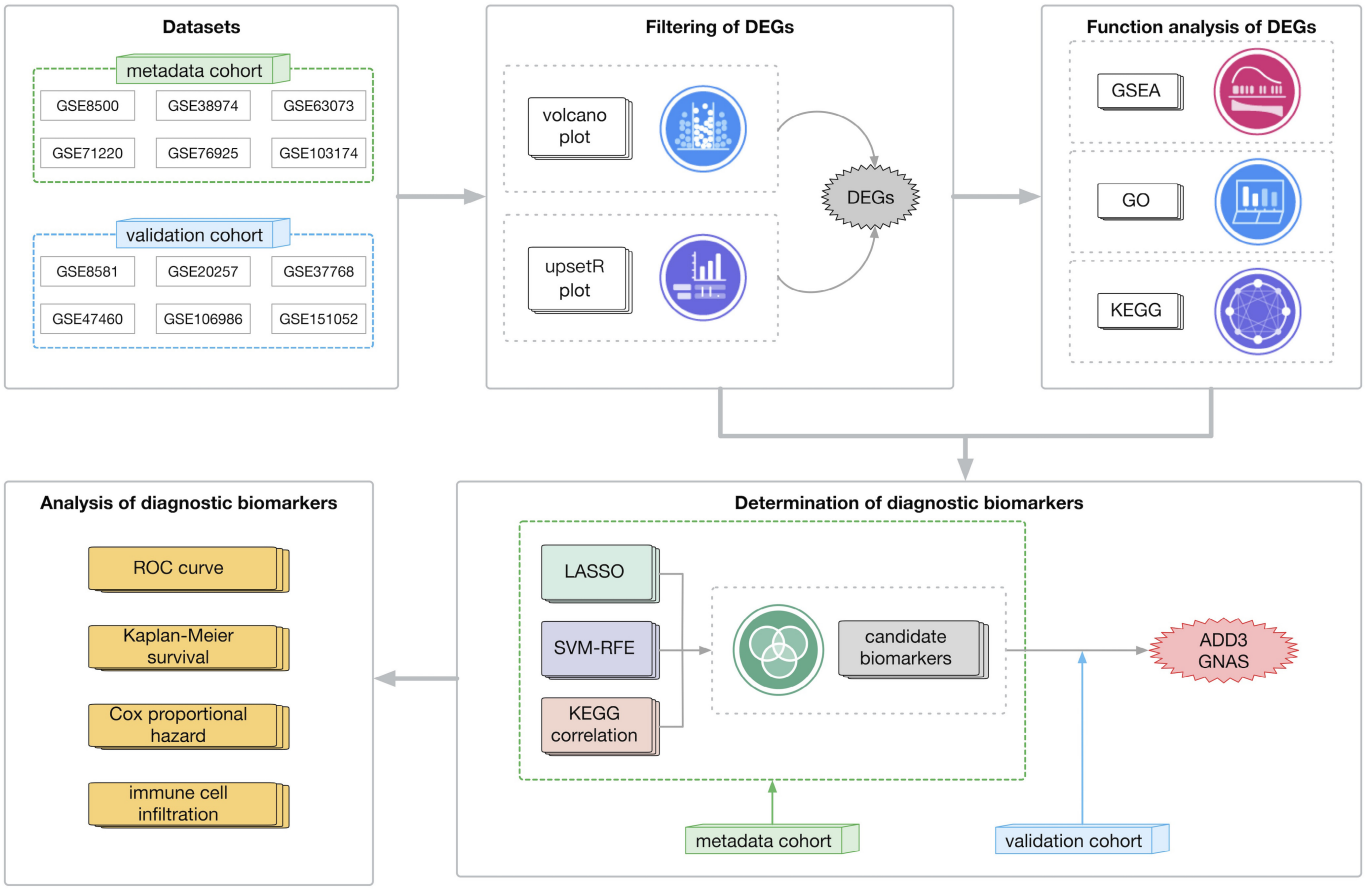
Overview of the study workflow. This flowchart illustrates the sequential methodology employed in the study, including the selected datasets, filtering of DEGs, functional analysis of DEGs, determination of diagnostic biomarkers and analysis of diagnostic biomarkers.

Figure 1 illustrates the overall pipeline of our study, including dataset selection, filtering of DEGs, functional analysis of DEGs, determination of diagnostic biomarkers and analysis of diagnostic biomarkers.

### 3.1 Filtering of DEGs

**FIGURE 2:**
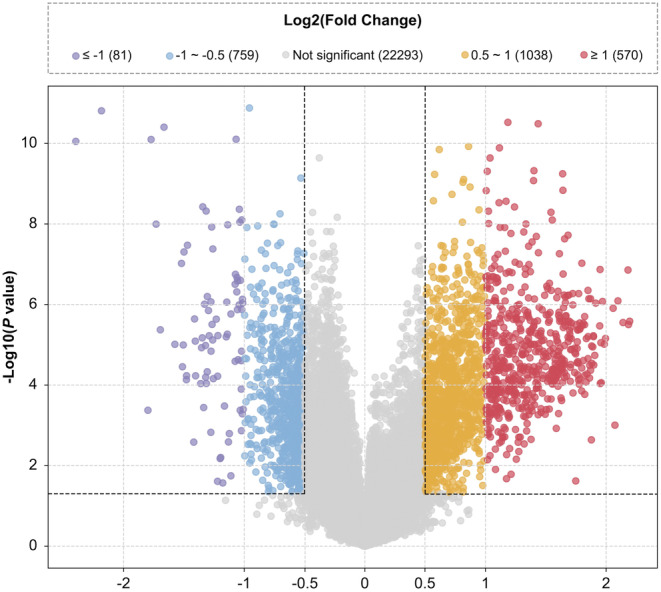
Volcano plot of DEGs. DEGs with log2FC ≤−1 are shown in purple, between −1 and −0.5 in blue, between 0.5 and 1 in orange, and ≥1 in red. Grey dots represent genes that are not significantly differentially expressed. The dashed lines indicate the significance threshold (*p* < 0.05) and fold change cutoffs.

**FIGURE 3:**
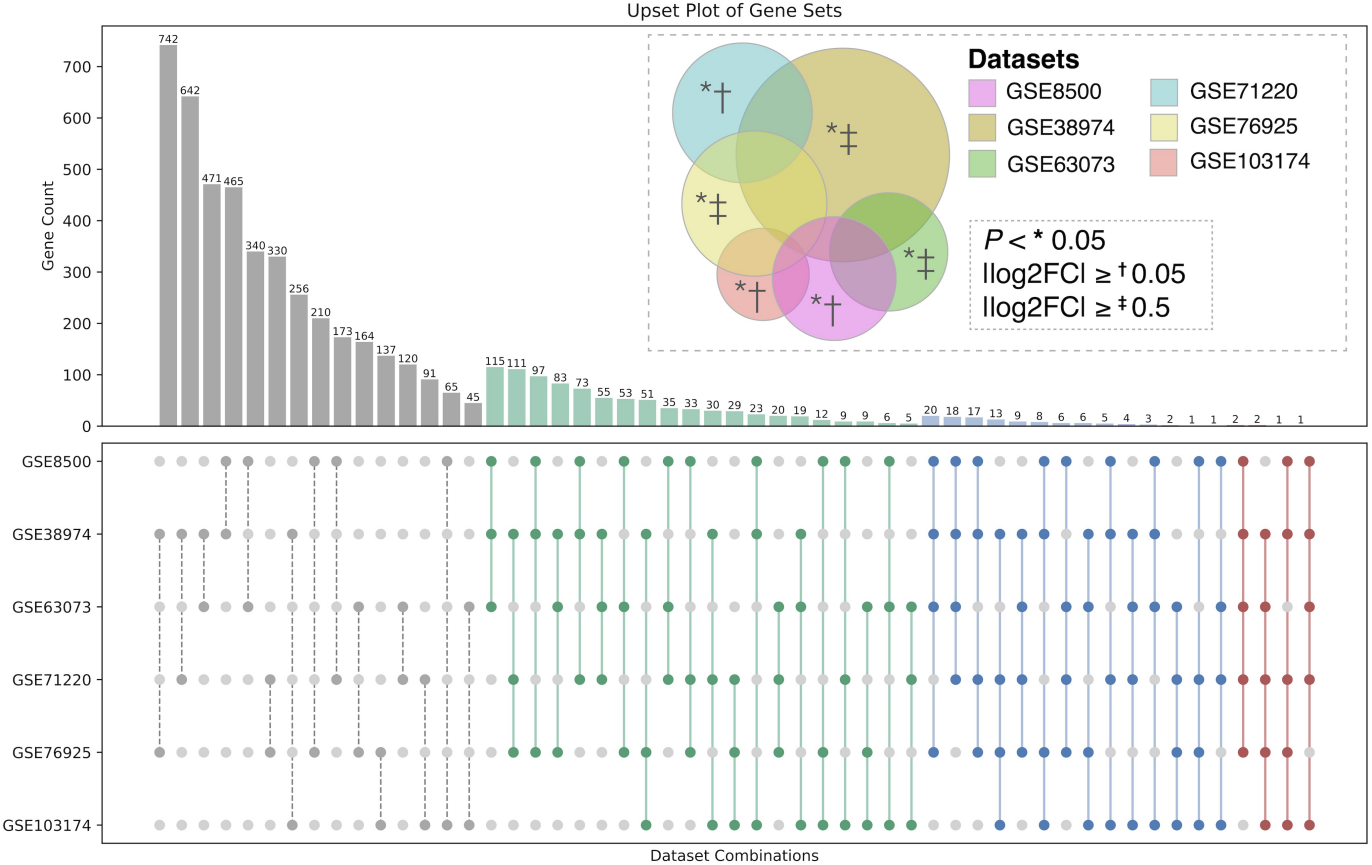
UpsetR plot of DEGs across six GEO datasets in the metadata cohort. DEGs shared by multiple datasets are represented with different colours: Grey, green, blue and red indicate intersections of 2, 3, 4 and 5 datasets, respectively. The vertical bars represent the number of DEGs in each intersection. A Venn diagram in the upper right illustrates the six datasets and indicates the different inclusion criteria used for each dataset.

Basic information of microarray datasets, including the metadata and validation cohorts, was shown in Table 1. DEGs with log2FC and *p*‐values among these cohorts were provided in supplementary files. The volcano plot revealed that there were 81 genes with log2FC ≤−1, 759 genes with log2FC between −1 and −0.5, 1038 genes with log2FC between 0.5 and 1 and 570 genes with log2FC ≥1 based on the selection criteria in the metadata cohort (Figure 2). Given the extensive data from the six datasets, we further refined the results and identified 987 DEGs, including 885 up‐regulated and 102 down‐regulated genes using an upsetR plot according to our selection criteria (Figure 3).

**TABLE 1:** Basic information of microarray datasets from GEO, including the metadata and validation cohorts.

Cohort type	Dataset	Platform	Age	Gender	Sample volume (COPD/controls)	Sample source	Smoking status		Authors	Country	Last update
							COPD	Control			
Metadata	GSE8500	GPL3991	62.77 ± 11.23	31 male 17 female	22/26	Lung tissue	All current	21 current 5 never	Wang et al.	USA	November 2014
	GSE38974	GPL4133	61.19 ± 9.82	24 male 8 female	23/9	Lung tissue	All current		Ezzie et al.	USA	February 2018
	GSE63073	GPL3991	Not mentioned		22/20	Lung tissue	All current		Stepaniants et al.	USA	April 2019
	GSE71220	GPL11532	63.57 ± 6.31	408 male 209 female	560/57	Blood	131 current 429 former	30 former 27 never	Obeidat et al.	Canada	November 2016
	GSE76925	GPL10558	63.94 ± 7.35	67 male 84 female	111/40	Lung tissue	All former		Morrow et al.	USA	May 2023
	GSE103174	GPL13667	66.15 ± 8.53	31 male 22 female	37/16	Lung tissue and blood	28 current 16 former	9 current 12 never	Cruz et al.	Spain	July 2021
Validation	GSE8581	GPL570	64.61 ± 9.65	14 male 19 female	15/18	Lung tissue	Not mentioned		Bhattacharya et al.	USA	March 2019
	GSE20257	GPL570	43.69 ± 9.88	95 male 40 female	23/112	Lung tissue	All current	53 current 59 never	Shaykhiev et al.	USA	March 2019
	GSE37768	GPL570	Not mentioned		18/20	Lung tissue	All current	11 current 9 never	Bastos et al.	Spain	March 2019
	GSE47460	GPL6480	62.63 ± 10.41	55 male 37 female	75/17	Lung tissue	3 current 66 former 3 never	15 former 8 never	Tedrow et al.	USA	October 2019
	GSE106986	GPL13497	66.84 ± 9.79	12 male 7 female	14/5	Lung tissue	10 current 4 former	All never	Heinbockel et al.	Germany	February 2018
	GSE151052	GPL17556	56.73 ± 10.78	Not mentioned	77/40	Lung tissue	Not mentioned		Xu et al.	Canada	August 2020

### 3.2 Function analysis of DEGs

**FIGURE 4:**
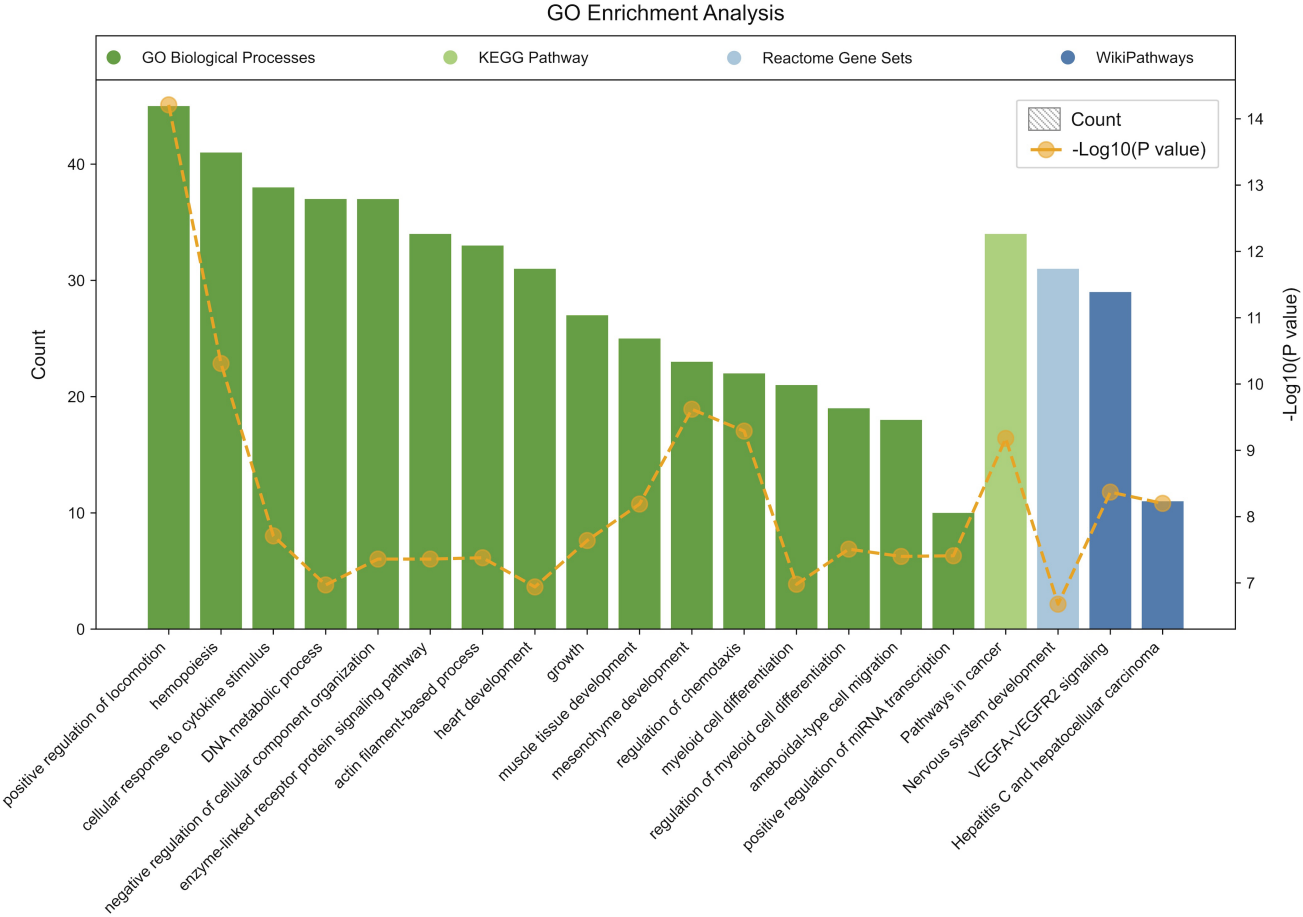
Bar chart of GO enrichment analysis of DEGs. GO biological processes, KEGG pathways, Reactome Gene Sets and Wiki pathways are represented in dark green, light green, light blue and dark blue, respectively. Each bar represents the count of enriched genes, with yellow dots indicating the −log10 (*p‐*value) for each category.

**FIGURE 5:**
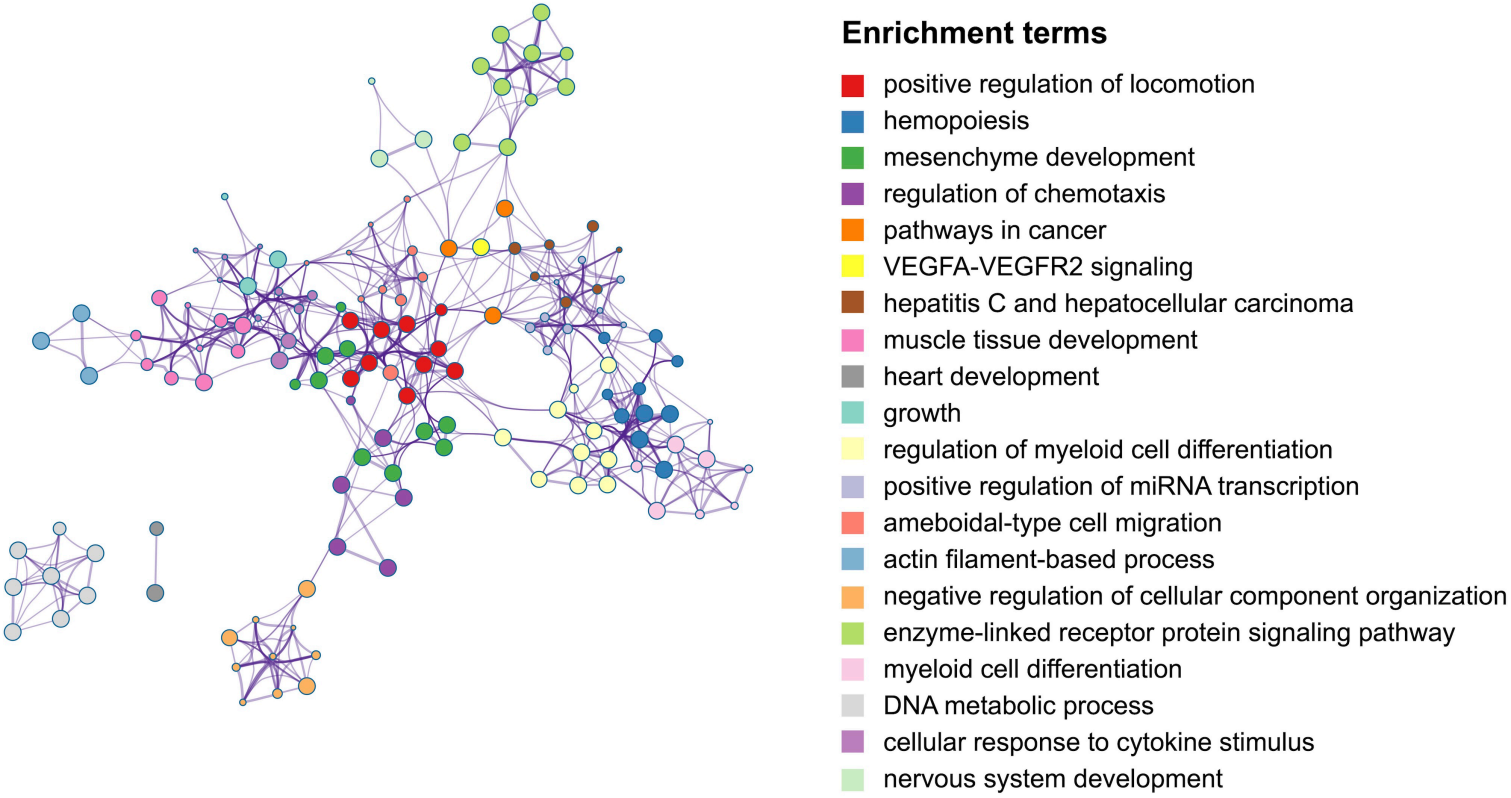
Network relationship plots among all enrichment terms. Each node represents an enriched term, and the edges indicate the relationships between these terms.

GO enrichment analysis demonstrated significant enrichment of DEGs across four categories, with a predominant focus on biological processes (BP), as illustrated in Figure 4. The top three enriched BP terms were positive regulation of locomotion, hemopoiesis and cellular response to cytokine stimulus. What's more, the interconnections between each enriched term are depicted in Figure 5, most terms are closely connected except for DNA metabolic process and heart development.

**FIGURE 6:**
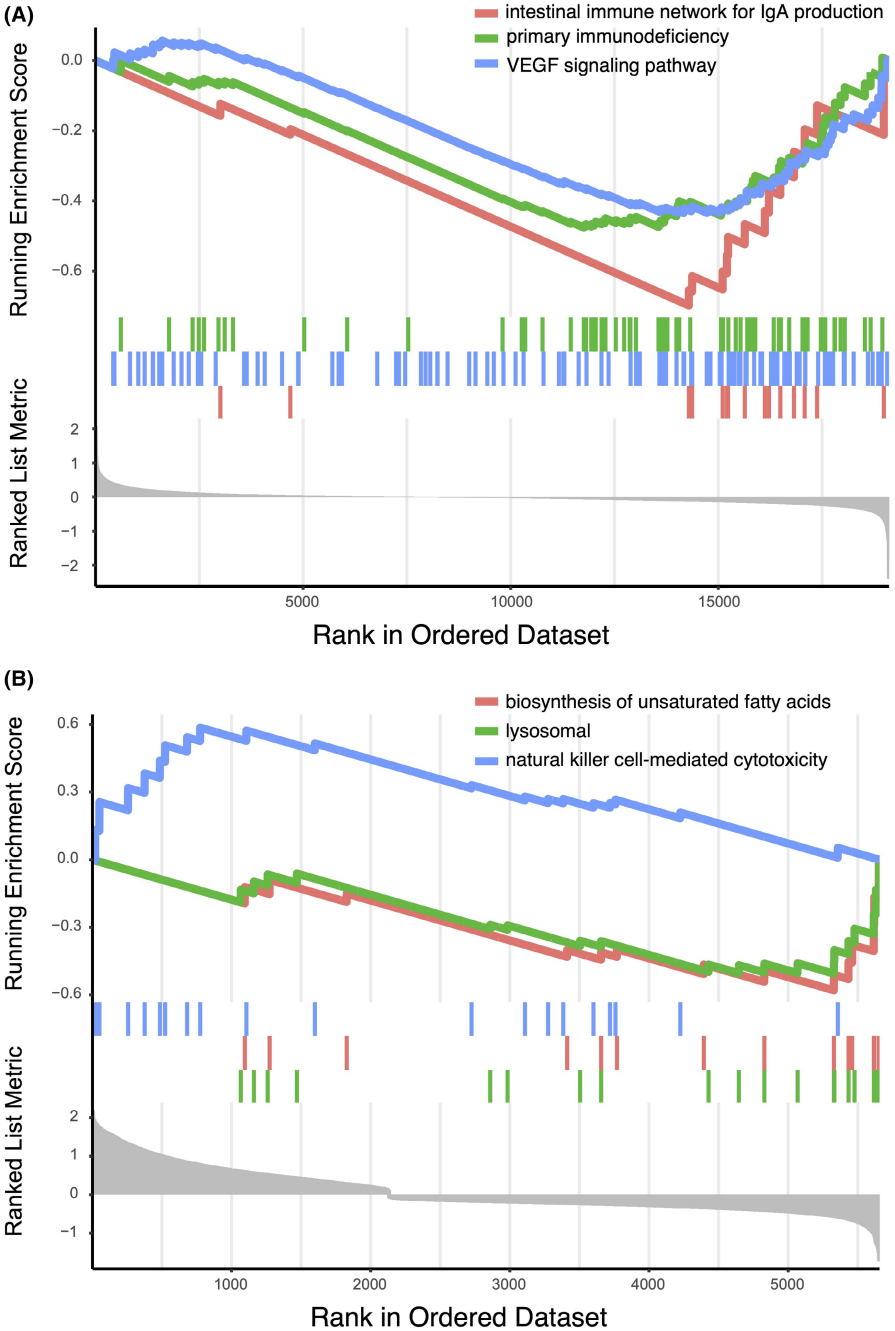
GSEA enrichment analysis of DEGs between COPD and controls. The *x*‐axis represents the rank in the ordered dataset, while the *y*‐axis displays the running enrichment score, indicating the degree of enrichment for each gene set within the dataset. (A) GSEA enrichment analysis results in COPD. (B) GSEA enrichment analysis results in controls.

GSEA results indicated that the intestinal immune network for IgA production, primary immunodeficiency and VEGF signalling pathway were the top three most significantly enriched terms in COPD samples (Figure 6A). Conversely, biosynthesis of unsaturated fatty acids, lysosome and NK cell‐mediated cytotoxicity were the top three most significantly enriched terms in controls (Figure 6B).

**FIGURE 7:**
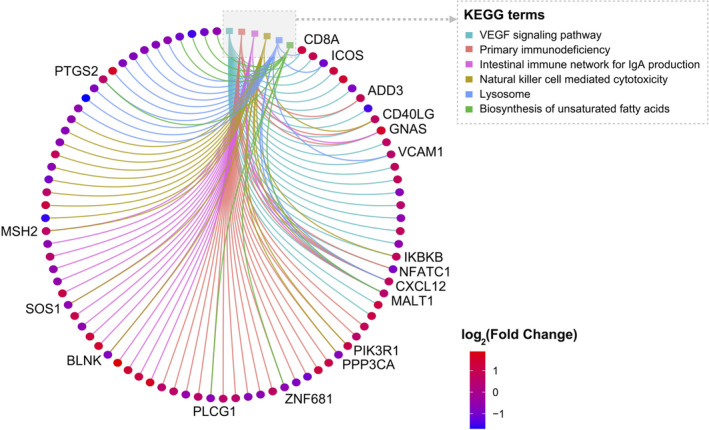
KEGG pathway‐gene correlation analysis. Genes are represented by circles and KEGG pathways by squares. A total of 83 genes are linked to various pathways, and genes associated with two or more pathways are explicitly labelled to highlight their multifunctional roles in the network.

Focusing on the top three pathways ranked in both the COPD and control groups from the GSEA results, KEGG pathway‐gene correlation analysis showed that a total of 83 genes were selected, revealing a complex network of interactions. Notably, among these genes, 18 were found to be associated with two or more pathways, highlighting their potential multifunctional roles in the BP under investigation (Figure 7).

### 3.3 Determination of diagnostic biomarkers

**FIGURE 8:**
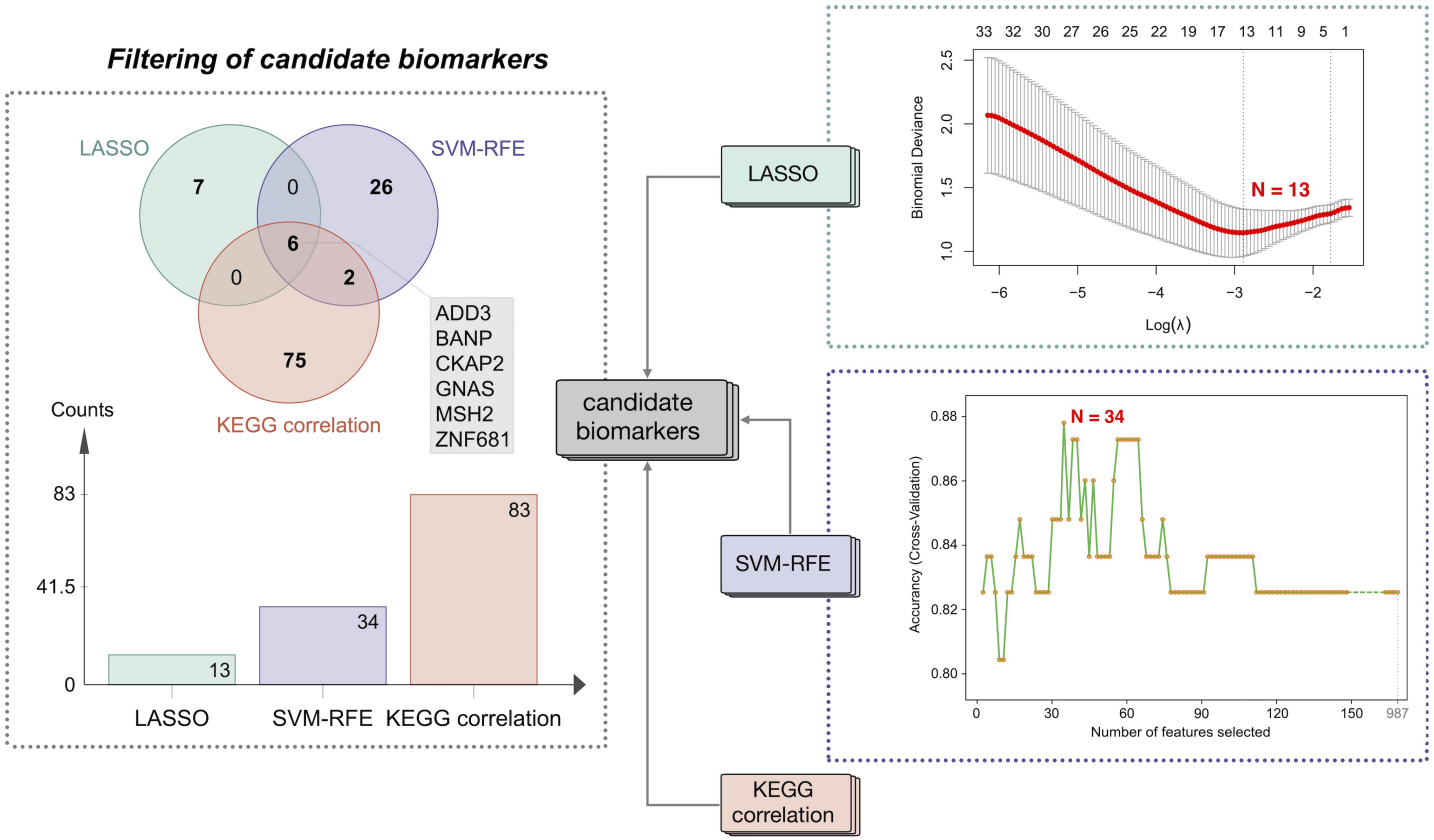
Flowchart of filtering candidate biomarkers. Candidate biomarkers were screened by the intersection of LASSO regression, SVM‐RFE and KEGG correlation analysis.

The process of filtering candidate biomarkers was shown in Figure 8. The LASSO logistic regression algorithm filtered out 13 meaningful variables, while the SVM‐RFE algorithm pinpointed 34 significant features. Combining the results of three methodologies including the LASSO, SVM‐RFE and KEGG correlation, the intersecting candidate biomarkers were ADD3, BANP, CKAP2, GNAS, MSH2 and ZNF681.

**FIGURE 9:**
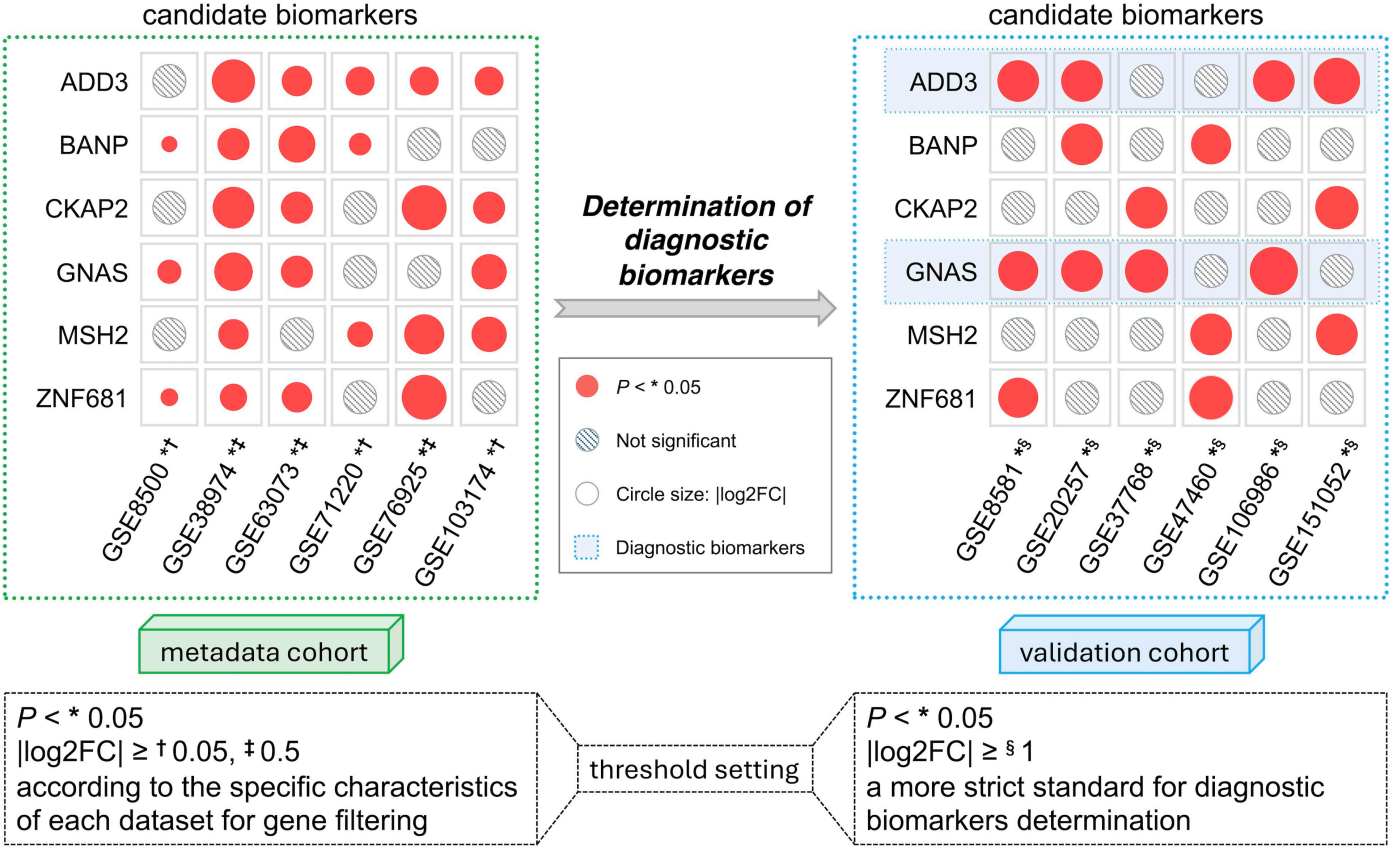
Flowchart of the determination of diagnostic biomarkers. The expression levels of six candidate biomarkers are shown in both the metadata cohort and validation cohort. Different threshold settings were applied for the two cohorts based on their respective purposes. Significant genes and their log2FC are indicated by red dots.

Subsequently, the expression levels of these variables in both the metadata and validation cohorts are shown in Figure 9. ADD3 and GNAS were upregulated in four different validation datasets, while BANP, CKAP2, MSH2 and ZNF681 were upregulated in only two different validation datasets. Finally, based on our inclusion criteria, ADD3 and GNAS were ultimately identified as our diagnostic biomarkers.

### 3.4 Clinical utility of ADD3 and GNAS

**FIGURE 10:**
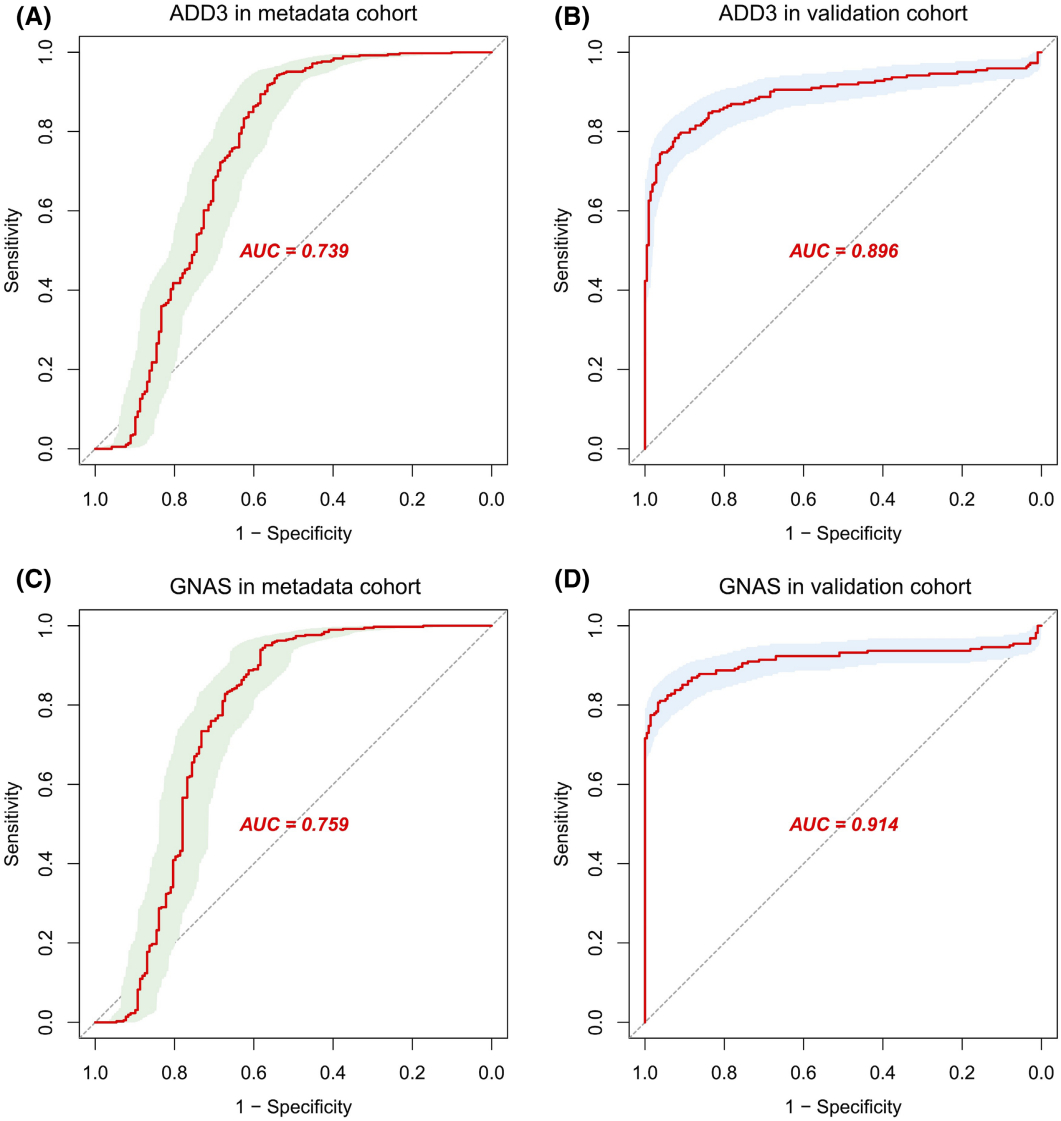
Validation of diagnostic efficacy of two diagnostic biomarkers. (A) ROC of ADD3 in the metadata cohort; (B) ROC of ADD3 in the validation cohort; (C) ROC of GNAS in the metadata cohort; (D) ROC of GNAS in the validation cohort.

We further assessed the diagnostic efficacy of the two specifically upregulated genes ADD3 and GNAS using ROC curves. The AUC for ADD3 in the metadata cohort and validation cohort were 0.739 (Figure 10A) and 0.896 (Figure 10B), respectively. Similarly, the AUC for GNAS in the metadata cohort and validation cohort were 0.759 (Figure 10C) and 0.914 (Figure 10D), respectively. These findings underscore their high diagnostic value for COPD.

**FIGURE 11:**
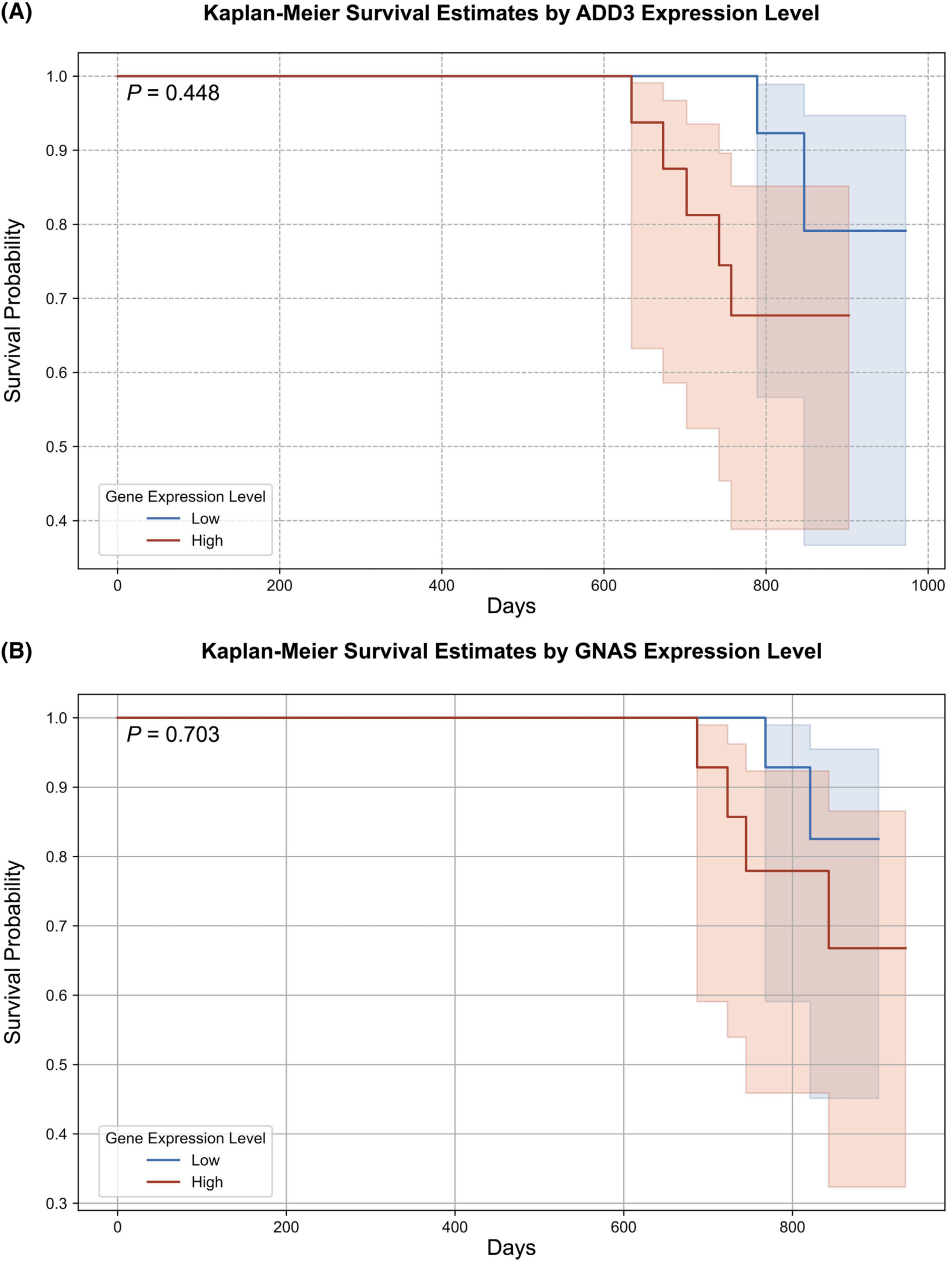
Kaplan–Meier survival estimates by expression level of diagnostic biomarkers on survival probability over time in COPD patients. (A) ADD3; (B) GNAS.

Furthermore, survival analysis using the Kaplan–Meier method revealed no statistically significant difference in OS between high and low expressions of ADD3 and GNAS in the COPD samples from the validation cohort, with *p*‐values of 0.448 (Figure 11A) and 0.703 (Figure 11B), respectively.

The results of the Cox proportional hazards regression analysis were shown in Table 2. The expression levels of ADD3 showed a positive correlation with increased hazard ratios, suggesting a higher risk associated with higher expression levels (coefficient = 1.22, hazard ratios = 3.40, *p* = 0.14). However, this association did not reach statistical significance. Similarly, GNAS expression was positively correlated with an increased hazard ratio (coefficient = 0.94, hazard ratios = 2.56, *p* = 0.28), yet these results were also not statistically significant. The concordance indexes were 0.71 for ADD3 and 0.65 for GNAS, indicating moderate predictive accuracy.

**TABLE 2:** The Cox proportional hazards regression analysis results for ADD3 and GNAS, detailing the coefficients, hazard ratios, *p*‐values, concordance indices, and partial AIC values, which elucidate their prognostic significance in COPD patients from the validation cohort.

Gene	Coefficient	Hazard ratios	*p*‐value	Concordance	Partial AIC
ADD3	1.22 (−0.42, 2.86)	3.40 (0.66, 17.51)	0.14	0.71	45.22
GNAS	0.94 (−0.76, 2.64)	2.56 (0.47, 14.00)	0.28	0.65	38.01

Abbreviation: AIC, akaike information criterion.

### 3.5 Overview of immune cell infiltration

**FIGURE 12:**
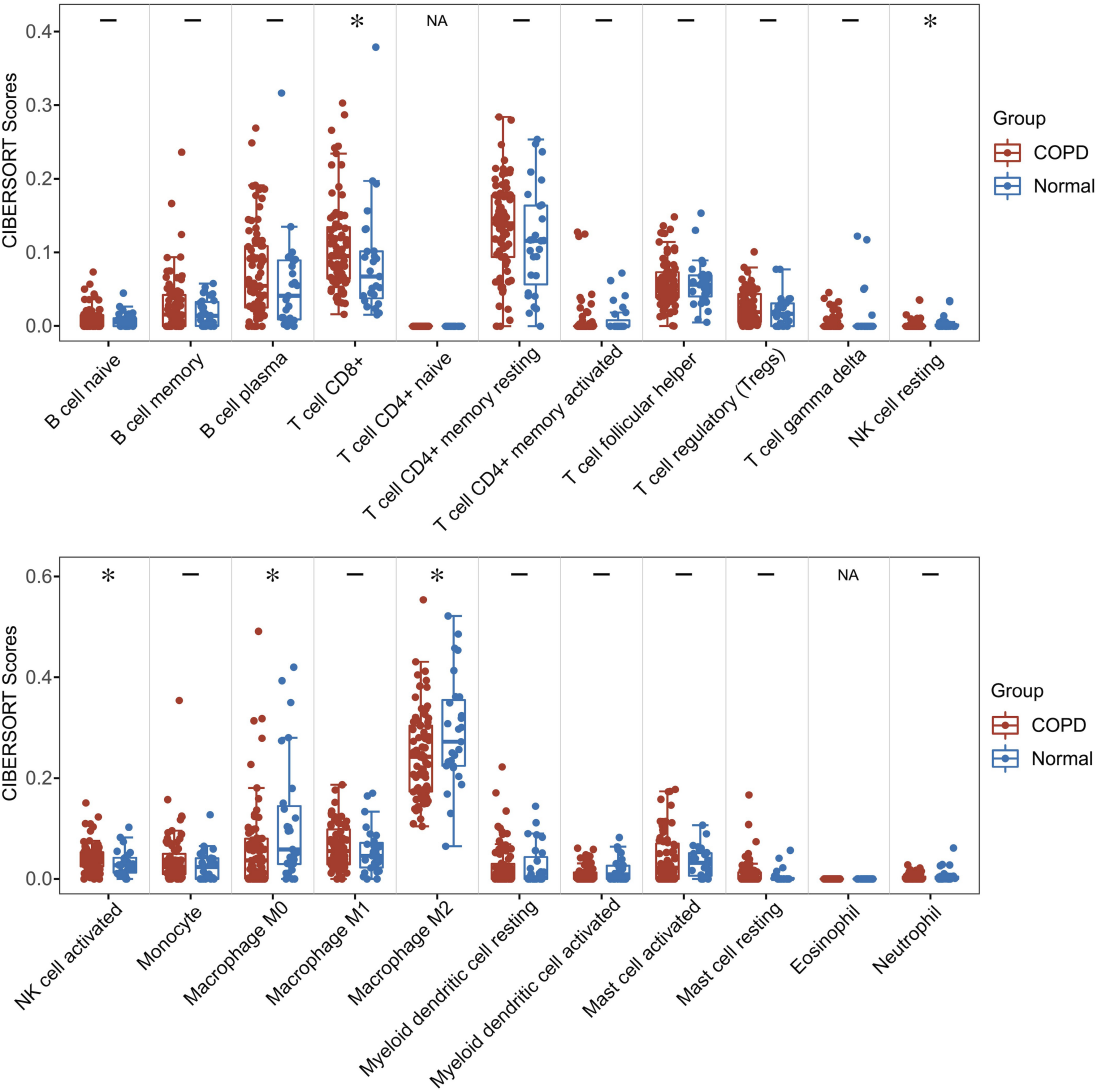
Box plot of the proportion of 22 types of immune cell infiltration between COPD and normal samples. Red in the boxes represents the COPD disease group and blue represents the normal group. *p < 0.05; –, not significant; NA, not available.

Our analysis uncovered significant differences in the infiltration levels of several immune cells. Specifically, T cell CD8^+^ (*p* = 0.025), resting NK cells (*p* = 0.032) and activated NK cells (*p* = 0.012) were significantly upregulated in COPD samples compared to healthy controls. Besides, control samples exhibited a higher degree of Macrophage M0 (*p* = 0.026) and Macrophage M2 (*p* = 0.049) infiltration compared to COPD samples. These results are illustrated in Figure 12.

### 3.6 Correlation analysis between ADD3, GNAS and infiltrating immune cells

**FIGURE 13:**
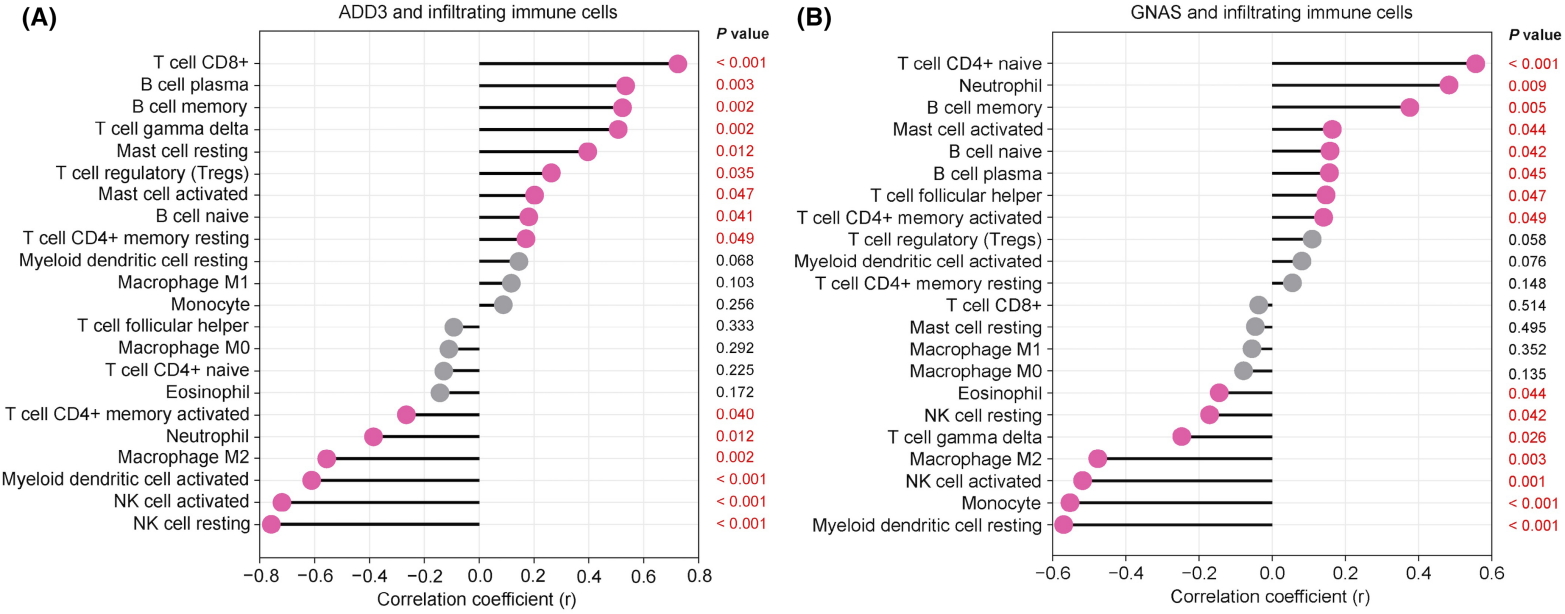
Correlation of two diagnostic biomarkers with 22 types of immune cell infiltration in COPD. (A) ADD3; (B) GNAS.

Furthermore, we visualized the relationship between the expression levels of our two diagnostic biomarkers, ADD3 (Figure 13A) and GNAS (Figure 13B), and the infiltration of 22 types of immune cells.

ADD3 was significantly positively associated with T cell CD8^+^ (*r* = 0.691), B cell plasma (*r* = 0.501) and B cell memory (*r* = 0.488), T cell gamma delta (*r* = 0.473), mast cell resting (*r* = 0.362), T cell regulatory (Tregs) (*r* = 0.230), mast cell activated (*r* = 0.168), B cell naive (*r* = 0.147) and T cell CD4^+^ memory resting (*r* = 0.137). And it was significantly negatively associated with NK cell resting (*r* = −0.736), NK cell activated (*r* = −0.686), myeloid dendritic cell activated (*r* = −0.577), macrophage M2 (*r* = −0.522), neutrophil (*r* = −0.352) and T cell CD4^+^ memory activated (*r* = −0.232).

GNAS was significantly positively associated with T cell CD4^+^ naive (*r* = 0.531), neutrophil (*r* = 0.458), B cell memory (*r* = 0.351), mast cell activated (*r* = 0.139), B cell naive (*r* = 0.133), B cell plasma (*r* = 0.131), T cell follicular helper (*r* = 0.122) and T cell CD4^+^ memory activated (*r* = 0.115). And it was significantly negatively associated with Myeloid dendritic cell resting (*r* = −0.545), monocyte (*r* = −0.528), NK cell activated (*r* = −0.494), macrophage M2 (*r* = −0.451), T cell gamma delta (*r* = −0.223), NK cell resting (*r* = −0.146) and eosinophil (*r* = −0.121).

## 4 DISCUSSION

As a chronic respiratory disease with significant global morbidity and mortality, COPD has garnered considerable interest due to the crucial importance of early diagnosis and intervention. Despite numerous therapeutic advances aimed at mitigating COPD symptoms, slowing disease progression and enhancing patient quality of life, early COPD diagnosis rates remain disappointingly low.^24^ Our study highlights that discovering and studying COPD biomarkers could enhance clinical understanding of the disease, potentially leading to improved diagnostic strategies.

The well‐documented involvement of immune cell infiltration in the pathogenesis and progression of COPD highlights its significance in this chronic respiratory disease.^7^, ^25^, ^26^ Consequently, identifying novel and effective diagnostic biomarkers for COPD among immune cell components is a promising research direction that could facilitate early intervention and improve clinical outcomes. DEGs have recently gained traction as potential biomarkers for respiratory diseases, particularly COPD.^8^, ^27^, ^28^ However, despite their promising role, few studies have explored the association between DEGs and immune infiltration in COPD. Therefore, we aim to uncover potential COPD diagnostic biomarkers and explore the role of immune infiltration in its pathophysiology.

This study introduces a novel retrospective analysis that extensively examines GEO datasets to uncover potential diagnostic biomarkers among DEGs and immune cell infiltration in COPD patients. Unlike conventional data inclusion methods that typically incorporate only 2 or 3 GEO datasets, we utilized both metadata and validation cohorts for the screening of candidate biomarkers and the determination of diagnostic biomarkers, respectively. In the metadata cohort, a more lenient |log2FC| threshold (≥0.05 or ≥0.5) was applied based on the specific characteristics of each dataset. For example, datasets like GSE8500 and GSE71220 exhibited gene expression profiles where nearly all genes had |log2FC| values below 0.5, and applying a stricter threshold in these cases would have resulted in limited gene selection, potentially compromising the breadth of our candidate pool. Conversely, in datasets like GSE38974, GSE63073 and GSE76925, where gene expression differences were more pronounced, we adopted a slightly stricter threshold (|log2FC| ≥0.5) to refine our results and avoid overwhelming the selection with a large number of DEGs. In the validation cohort, our focus shifted to the determination of diagnostic biomarkers. Therefore, we applied a more stringent threshold (|log2FC| ≥1) to ensure that the candidate biomarkers selected had robust expression differences, reflecting a higher level of confidence in their diagnostic potential.

Additionally, our innovative data inclusion method incorporated up to six GEO datasets, considering genes appearing in at least three datasets. This methodology, using an incomplete intersection of datasets, is rarely reported yet demonstrates remarkable efficiency in managing extensive datasets.^18^, ^19^ It accounts for variability across different datasets and capitalizes on their consensual features, thereby minimizing integration errors and enhancing the credibility of findings. Using this method, we initially identified 987 DEGs, including 885 up‐regulated and 102 down‐regulated, which appeared in 3–5 different datasets within the metadata cohort. However, no single DEG was identified across all six cohorts. This variation can be attributed to several factors, including differences in clinical subtypes of COPD, variations in patient demographics, sample collection methods and experimental conditions across the datasets. These factors can influence gene expression profiles and result in variability in DEGs identified across different cohorts.

Subsequently, a comprehensive functional analysis was conducted on the DEGs identified. Initially, we utilized Metascape, a robust bioinformatics tool that extends beyond traditional GO analysis to include various biological databases and enrichment pathways, including Wiki pathways, KEGG pathways and Reactome Gene Sets. We focused specifically on the GO terms, and it revealed that these DEGs were predominantly involved with positive regulation of locomotion, hemopoiesis, cellular response to cytokine stimulus and among others. The enrichment in the positive regulation of locomotion suggests changes in cell motility critical for tissue remodelling and repair, often disrupted in disease states.^29^ Significant enrichment in hemopoiesis underscores the role of bone marrow cells in normal and disease physiology, potentially affecting immune and inflammatory responses.^30^, ^31^ Lastly, the involvement of cellular response to cytokine stimulus highlights the importance of cytokine signalling in modulating immune reactions and inflammation.^32^

GSEA results showed that intestinal immune network for IgA production, primary immunodeficiency and VEGF signalling pathway were the top three most significantly enriched terms in COPD samples, corroborating known factors associated with the disease. The enrichment of the intestinal immune network for IgA production in COPD samples is particularly intriguing. While IgA primarily protects mucosal surfaces, its systemic effects in respiratory diseases are increasingly studied.^33^ Previous studies have indicated crosstalk between gut and lung immunity, known as the gut‐lung axis, suggesting mutual influences on gut and lung health.^34^ Our findings indicate that further research into this axis could reveal new mechanisms of COPD pathogenesis and therapeutic targets. Primary immunodeficiency being highlighted in our analysis suggests an underlying vulnerability in the immune system of individuals with COPD.^35^, ^36^ This aligns with the notion that impaired immune responses exacerbate COPD severity and progression, underscoring the need for deeper understanding of immune dysregulation in these patients.^37^ Additionally, the enrichment of the VEGF signalling pathway aligns with its role in promoting angiogenesis and vascular permeability. In COPD, this may relate to pathological vascular remodelling, contributing to chronic inflammation and tissue damage. Prior research has identified aberrant VEGF signalling in COPD, corroborating our results and suggesting therapeutic potential through pathway modulation.^38^, ^39^

Moreover, GSEA results identified biosynthesis of unsaturated fatty acids, lysosomal and NK cell‐mediated cytotoxicity as the top three enriched pathways in the healthy group. The biosynthesis pathway of unsaturated fatty acids, known for its anti‐inflammatory properties, suggests a role in maintaining pulmonary homeostasis, potentially reducing inflammation and improving lung function through diet.^40^ Essential for cellular homeostasis, the lysosomal pathway degrades and recycles waste, preventing buildup that could trigger inflammation. In COPD, its impairment may enhance oxidative stress and airway inflammation.^41^, ^42^ NK cells are crucial in the innate immune system's early defence against infections. Their active pathways in controls highlight their role in immune surveillance and preventing infections that could worsen chronic lung diseases.^43^ In COPD, dysfunction of NK cells may contribute to disease progression by inadequately controlling initial infections.^13^

However, the primary objective of GSEA was to identify pathways showing significant differences between diseased and normal states. This method assessed the overall performance of gene sets rather than focusing on individual gene expression differences. Based on this, our subsequent KEGG pathway‐gene correlation analysis offered a detailed examination of individual gene expressions within these pathways. This approach enabled us to pinpoint specific gene expression changes under defined conditions and map these alterations within particular KEGG pathways The pathway‐gene network results revealed that 83 genes were enriched in the top three pathways identified by GSEA, with 18 genes enriched in two or more pathways. This indicates the multifunctional roles of these genes, suggesting their involvement in multiple BP pivotal to the disease.

The integration of machine learning techniques, specifically LASSO regression and SVM‐RFE, strategically refined DEGs by reducing data dimensionality and enhancing selection precision through isolating the most informative predictors. Crucially, merging these machine learning outcomes with our initial functional analyses yielded six candidate biomarkers. This convergence of these three methodologies validated the relevance of genes and reinforced our confidence in their biomarker potential.

Subsequently, two upregulated diagnostic biomarkers, ADD3 and GNAS, were confirmed using the validation cohort, which, like the metadata cohort, included up to six datasets, demonstrating robust expression patterns consistent with their proposed roles in COPD. Although only four out of the six validation cohorts showed significant results for these biomarkers, the non‐significant cohorts still exhibited relatively high log2FC values. ADD3 had log2FC values of 0.87 and 0.90 in GSE37768 and GSE47460, respectively, while GNAS had log2FC values of 0.88 and 0.93 in GSE47460 and GSE151052, respectively. These expression changes, though not meeting our stringent significance threshold, remain biologically relevant. Besides, the small sample sizes in the non‐significant cohorts minimized their impact on the overall AUC values, which were 0.896 for ADD3 and 0.914 for GNAS when including all six validation datasets. What's more, the moderate decrease in AUC values observed in the metadata cohort may be attributed to differences in tissue or blood origins across the datasets.

In the survival analysis, we conducted the Kaplan–Meier survival curves and the Cox proportional hazards model, using survival expression data from the GSE8581, GSE37768 and GSE106986 in the validation cohort. Unfortunately, survival data from other datasets were not available in public databases at this time. Firstly, the Kaplan–Meier survival curves showed no statistically significant differences in OS between expression levels of ADD3 and GNAS in COPD patients, which may be attributed to the limited sample size. Besides, since COPD primarily affects elderly individuals, causes of death may not be directly related to COPD, especially following standardized pharmacological management, which could increase OS rates. Additionally, the Cox proportional hazards model indicated that ADD3 and GNAS may serve as potential risk factors exacerbating disease progression. Although the results were not statistically significant, they align with our functional analysis that identifies both genes as facilitators in COPD progression. This insight directs future research to explore these biomarkers as potential targets for therapeutic intervention and risk stratification in COPD. Above all, we propose that ADD3 and GNAS could play pivotal roles in modulating the onset and progression of COPD.

ADD3 (Adducin 3) functions primarily in the regulation of the cytoskeleton, influencing cell shape and stability, which are critical in maintaining airway structure and function,^44^ this role extends to the modulation of blood pressure and potentially inflammatory responses in vascular and airway tissues.^45^ GNAS (GNAS Complex Locus), known for its complex imprinted expression pattern, plays a key role in classical signal transduction pathways, affecting various cellular responses, including hormonal stimuli, this involvement underscores its potential impact on the inflammatory and immune responses typical of COPD.^46^, ^47^

Besides, our functional analysis revealed that ADD3 is upregulated and enriched in two pathways, including the primary immunodeficiency pathway and the VEGF signalling pathway. Similarly, GNAS is upregulated and enriched in three pathways, including the primary immunodeficiency pathway, the VEGF signalling pathway, and the intestinal immune network for IgA production pathway. The pathways involving ADD3 and GNAS closely relate to COPD, reflecting the enriched pathways identified in the COPD group. These findings suggest potential mechanistic links between two genes and COPD pathogenesis.

COPD is characterized by progressive airflow limitation that impairs lung function, primarily manifesting as emphysema and chronic bronchitis.^48^ Inflammation is a primary driver of COPD pathogenesis, with various irritants triggering immune cell infiltration and the release of mediators that lead to tissue destruction and a progressive decline in lung function.^49^ Our application of the CIBERSORT evaluation provided insights into the profiles of immune cell infiltration in COPD samples compared to healthy counterparts, revealing a complex interplay of immune cell subtypes associated with the disease. We observed an increased infiltration of T cell CD8^+^, resting NK cells and activated NK cells in COPD tissues, while the proportions of macrophage M0 and M2 were notably diminished. This diversity of immune cells underscores the inflammatory landscape characteristic of COPD.

Further analysis showed that the overexpression of ADD3 and GNAS was significantly correlated with various immune cells, highlighting their complex roles in modulating immune responses. ADD3 and GNAS were positively correlated with B cell subsets (naive, memory, and plasma), indicating their involvement in humoral immunity, crucial in COPD. These associations may reflect their roles in enhancing antibody‐mediated responses, potentially worsening inflammation and tissue damage in COPD from persistent infections. Conversely, their negative correlations with NK cells (resting and activated) suggest an inhibitory effect on this critical component of innate immunity, possibly compromising viral defences and exacerbating COPD progression.

The divergent correlations between ADD3 and GNAS with T cell subsets and neutrophils highlight their distinct influences on adaptive and innate immune responses. ADD3's positive correlation with T cell gamma delta and negative association with neutrophils suggest it modulates inflammation and adaptive immunity, potentially enhancing mucosal defences and reducing acute inflammatory responses. Conversely, GNAS's positive correlation with neutrophils and negative correlation with T cell gamma delta may promote inflammatory processes during COPD exacerbations, consistent with its pro‐inflammatory effects observed in other pathologies.

The unique correlations with T cells, dendritic cells and monocytes further elucidate the nuanced roles of these genes in the pathophysiology of COPD. For example, ADD3's negative correlation with activated myeloid dendritic cells may suggest suppression of antigen presentation, potentially impacting immune competence. Meanwhile, GNAS's interactions suggest a potential to alter early immune activation pathways, which could influence the severity and frequency of COPD exacerbations.

These insights into the roles of ADD3 and GNAS in immune regulation provide a basis for developing therapeutic strategies to modulate these pathways and manage COPD progression. Further research, including the application of single‐cell RNA sequencing to validate CIBERSORT results, as well as the incorporation of additional survival data and updated GEO datasets, is needed to further substantiate the biological significance of these diagnostic biomarkers in COPD, potentially leading to novel interventions.

## 5 CONCLUSION

In conclusion, we identified that ADD3 and GNAS are significant diagnostic biomarkers through the comprehensive method based upon microarray datasets of COPD. CD8^+^ T cell, resting NK cell, activated NK cell and macrophage are salient immune factors to drive COPD pathogenesis. Elucidating the intricate mechanisms underlying aberrant immune cell infiltration in COPD represents a pivotal area for future research and is likely to illuminate promising immunotherapeutic targets for ameliorating disease progression. Given the putative roles of ADD3 and GNAS in perpetuating immune dysregulation, these molecules may have utility as personalized therapeutic targets, in addition to their potential as diagnostic biomarkers.

## AUTHOR CONTRIBUTIONS

**Zirui Zhu:** Conceptualization (lead); formal analysis (lead); methodology (lead); writing – original draft (equal). **Zhuo Zeng:** Investigation (lead); data curation (lead). **Baichen Song:** Visualization (lead). **Huishan Chen:** Writing – original draft (equal). **Huiqing Zeng:** Supervision (lead); writing – review and editing (lead).

## CONFLICT OF INTEREST STATEMENT

The authors confirm that there are no conflicts of interest.

## Supporting information


Appendix S1.

Appendix S2.

Appendix S3.

Appendix S4.

Appendix S5.

Appendix S6.

Appendix S7.

Appendix S8.

Appendix S9.

Appendix S10.

Appendix S11.

Appendix S12.

Appendix S13.

## Data Availability

DEGs with log2FC and *P* values among metadata and validation cohorts were provided in supplementary files, other data generated and analysed during the current study are available from the corresponding author on reasonable request.
